# BanglaSER: A speech emotion recognition dataset for the Bangla language

**DOI:** 10.1016/j.dib.2022.108091

**Published:** 2022-03-22

**Authors:** Rakesh Kumar Das, Nahidul Islam, Md. Rayhan Ahmed, Salekul Islam, Swakkhar Shatabda, A.K.M. Muzahidul Islam

**Affiliations:** a Department of Computer Science and Engineering, Stamford University Bangladesh, Bangladesh; b Department of Computer Science and Engineering, United International University, Bangladesh

**Keywords:** Speech emotion recognition, Sound processing, Deep Learning, Bangla language

## Abstract

The speech emotion recognition system determines a speaker's emotional state by analyzing his/her speech audio signal. It is an essential at the same time a challenging task in human-computer interaction systems and is one of the most demanding areas of research using artificial intelligence and deep machine learning architectures. Despite being the world's seventh most widely spoken language, Bangla is still classified as one of the low-resource languages for speech emotion recognition tasks because of inadequate availability of data. There is an apparent lack of speech emotion recognition dataset to perform this type of research in Bangla language. This article presents a Bangla language-based emotional speech-audio recognition dataset to address this problem. BanglaSER is a Bangla language-based speech emotion recognition dataset. It consists of speech-audio data of 34 participating speakers from diverse age groups between 19 and 47 years, with a balanced 17 male and 17 female nonprofessional participating actors. This dataset contains 1467 Bangla speech-audio recordings of five rudimentary human emotional states, namely angry, happy, neutral, sad, and surprise. Three trials are conducted for each emotional state. Hence, the total number of recordings involves 3 statements × 3 repetitions × 4 emotional states (angry, happy, sad, and surprise) × 34 participating speakers = 1224 recordings + 3 statements × 3 repetitions × 1 emotional state (neutral) × 27 participating speakers = 243 recordings, resulting in a total number of recordings of 1467. BanglaSER dataset is created by recording speech-audios through smartphones, and laptops, having a balanced number of recordings in each category with evenly distributed participating male and female actors, and would serve as an essential training dataset for the Bangla speech emotion recognition model in terms of generalization. BanglaSER is compatible with various deep learning architectures such as Convolutional neural networks, Long short-term memory, Gated recurrent unit, Transformer, etc. The dataset is available at https://data.mendeley.com/datasets/t9h6p943xy/5 and can be used for research purposes.

## Specifications Table

**Table:** 

Subject	Signal Processing
Specific subject area	Speech emotion recognition, Sound analysis
Type of data	Digital audio files
How the data were acquired	Bangladeshi voice data are recorded using the smartphone's default recording application, laptop, and microphone. Recordings are set to 3 to 4 s of duration and surrounding noises are removed using the Audacity software.Tools used: • Smartphone • Microphone • Headset • Asus GL503GE Laptop • Audacity software
Data format	Waveform Audio File Format (WAV).
Description of data collection	The speech-audio have been recorded in different categories: • Five different emotional states (Angry, Happy, Neutral, Sad, Surprise). • Using three different Bengali sentences: i. English 1: It's twelve o'clock English 2: It's twelve o'clock already! ii. English: I knew something like this would happen. iii. ? English: What kind of gift is this? The 1467 speech-audio recordings are collected from various groups of people from different groups of lives from different parts of Bangladesh aged between 19 to 47 years. 17 male and 17 female participating actors are chosen to maintain the balance between male and female speakers. These emotional expressions were simulated and enacted. Actors were given a detailed briefing about all the scripted statements and emotional states needed to come out of the recordings. The categorical emotional states were evaluated through fifteen human validators. Human recognition performance of the intended emotion is 80.5% (approx.) for the BanglaSER speech-audio dataset.
Data source location	City: Dhaka Country: Bangladesh
Data accessibility	**Repository name**: BanglaSER: A Bangla speech emotion recognition dataset. **Digital object identifier**: 10.17632/t9h6p943xy.5 **URL** to data: https://data.mendeley.com/datasets/t9h6p943xy/5

## Value of the Data

• The presented dataset is significant because it is only the second open-source speech-audio dataset in the Bangla language.
• This dataset can be utilized to train the machine learning models for speech emotion recognition (SER) task with proper generalization.
• This dataset can help researchers investigate a person's emotion in the Bangla language in diverse research domains of clinical studies, audio surveillance, human-computer interface, and home automation [1].
• It is a balanced dataset in terms of speech-audio recordings in each emotional category and an equal number of participating male and female actors across a diverse age group.
• Different audio features such as spectral and rhythmic features can be investigated from this dataset and applied to a variety of machine learning models to improve the SER performance in the low-resource Bangla language.

## 1 Data Description

BanglaSER is a Bangla speech-audio dataset, which is mainly developed for the SER task. BanglaSER contains 1467 speech-audio recordings of five rudimentary emotional states which are perceived as more natural than reading renditions [2]. The phrase ``perceived emotions'' relates to the extent to which the selected emotion matched the actor's intended emotion [3], thereby validating the corresponding labeling of the BanglaSER utterances. These emotional states are angry, happy, neutral, sad, and surprise. The BanglaSER contains 34 nonprofessional actors (17 male, 17 female), enunciating three lexically-matched Bangla statements in Bengali accent. Three trials were conducted for each emotional state. BanglaSER contains 1467 speech-audio files: three (3) sentences × three (3) repetitions × four (4) emotional states (angry, happy, sad, surprise) × 34 participating speakers = 1224 recordings/utterances + 243 speech-audio files: three (3) sentences × three (3) repetitions × one (1) emotional state (neutral) × 27 participating speakers = 243 recordings/utterances, resulting in a combined total of 1467 Bangla speech-audio recordings. The three sentences are i.  (“It's twelve o'clock” or “It's twelve o'clock already!”), ii.  (I knew something like this would happen), and iii. ? (What kind of gift is this?).

**Fig. 1:**
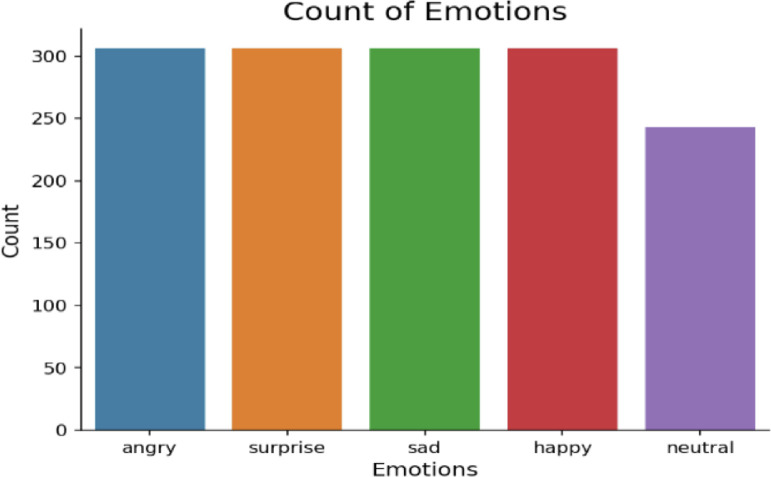
Class-wise speech-audio recording distribution in the BanglaSER dataset.

**Fig. 2:**
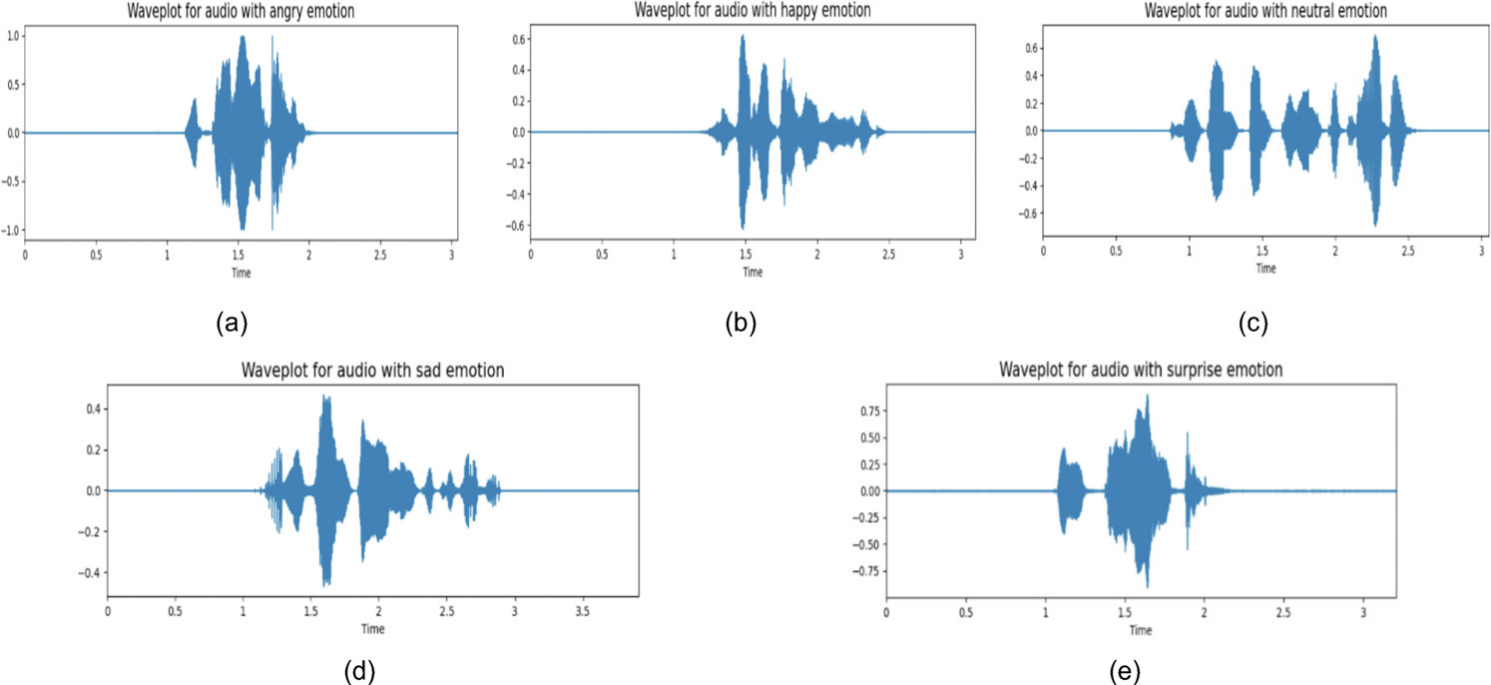
Sample waveform plot of a randomly selected sample of every emotional state, (a) angry, (b) happy, (c) neutral, (d) sad, and (e) surprise emotions of the BanglaSER dataset.

There are 34 individual folders in this dataset. Each folder represents a participant and contains speech-audio recordings of each actor. BanglaSER is both class and gender-balanced dataset with 306 recordings for angry, happy, sad, surprise emotions, and 243 recordings for the neutral emotion, as depicted in Fig. 1. The duration of each of the audio recordings is from 3 to 4 s. The audio files are recorded in 32-bit float WAV format. A waveform plot for every emotion of a randomly selected sample from the BanglaSER dataset is presented in Fig. 2. Here, the X-axis is representing different time frames measured in seconds, and the Y-axis represents amplitude that indicates the amount of air compression (> zero) or rarefaction (< zero) induced by a moving object, such as the vocal cords, and pressure equilibrium point (= zero) denotes silence. The typical range of the Y-axis is [1, -1]. However, we did not perform that scaling here so that we can visualize the waveforms clearly with auto-scaling of the python librosa library. Using the same scale for all the samples with different amplitudes will not be visually comparable which is why auto-scale is used for visualization purposes only. The features generated from the associated audio-recording of each waveform can be eventually scaled to a normalized value to train a machine learning-driven SER model. Since the number of speech-audio recordings is not very large in the BanglaSER dataset, various data augmentation methods such as adding additive white gaussian noise, time-shifting, pitch scaling, time-stretching, etc. can be applied to the dataset to expand the dataset and also improve the generalization of the machine-learning driven SER system. Several studies [3,4] have utilized various data augmentation techniques and improved the performance of SER systems as well as generic audio classification tasks.

**Fig. 3:**
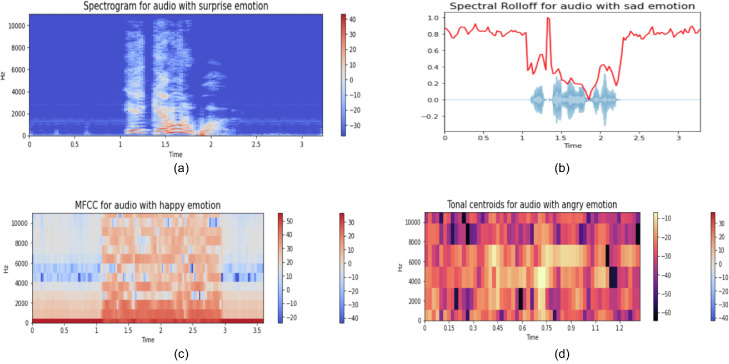
Sample (a) Mel-spectrograms, (b) Spectral Rolloff, (c) MFCCs, (d) tonal centroids of every category of emotions from the BanglaSER dataset.

There are several SER datasets available in literature covering English, Greek, Korean, and German languages, such as IEMOCAP [5], RAVDESS [6], MSP-IMPROV [2], AESDD [7], SAVEE [8], CADKES [9], and EMO-DB [10]. However, there is only one dataset for the SER task in the Bangla language, namely SUBESCO [11]. A simple comparison of these public SER datasets with BanglaSER is shown in Table 1. Several studies [12,13] have utilized those datasets and extracted acoustic features such as mel-frequency cepstral coefficients (MFCCs), tonal centroid, zero-crossing rate, spectral centroid, spectral roll-off, root-mean-square, mel-scaled spectrogram, and chromagram. By normalizing these features, we can feed them to the machine learning model as an input for the SER task. Some of the extracted mel-scaled spectrogram, MFCCs, tonal centroids, spectral roll-off feature from the BanglaSER dataset are presented in Fig. 3, where X-axis represents different time frames measured in seconds and Y-axis represents frequency. Some of the useful features along with their values and data range, appropriate for the proposed BanglaSER dataset to be utilized in the machine learning-driven SER task is provided in Table 2. We also provide a descriptive summary of the BanglaSER dataset in Table 3.

**Table 1:** A comparative summary among different public SER datasets and BanglaSER dataset (- Not mentioned, ✔- Yes, x - No).

Specifications	IEMOCAP	RAVDESS	MSP-IMPROV	EMO-DB	SAVEE	AESDD	CADKES	SUBESCO	**BanglaSER**
# scripted / target audio records	5255	1440	620	535	480	500	6760	7000	1467
# of speech-audio emotions	9	8	4	7	7	5	5	7	5
# of target sentences	3	2	15	10	15	19	52	10	3
# of participating actors	10	24	12	10	4	5	26	20	34
Professional actors	✔	✔	✔	✔	-	✔	x	✔	x
Sampling Rate	16 kHz	48 kHz	48 kHz	16 kHz	44.1 kHz	-	16 kHz	48 kHz	44.1 kHz
Class balance	✔	x	✔	x	x	✔	✔	✔	✔
Gender balance	✔	✔	✔	✔	x	x	✔	✔	✔
Language	English	English	English	German	English	Greek	Korean	Bangla	**Bangla**

**Table 2:** A suggested feature vector set for a machine-learning model to evaluate the BanglaSER dataset for the SER task.

Domain Name	Number of Features	Parameters
Time	3	Amplitude envelope (frame size = 1024, hop length = 512) Root mean square energy and Zero-crossing rate.
Frequency	145	Band energy ratio (split frequency = 2000, sample rate = 22050) Spectral centroid, Spectral bandwidth, Spectral rolloff (rate: 85), Tonal centroid, Chromagram (n_chroma = 12) and Mel-scaled spectrogram (n_mels = 128).
Cepstral	20	MFCCs (coefficients: 1-20, Discrete cosine transform type = 2).
Statistics	3	Entropy, Kurtosis, and Skewness.

**Table 3:** A descriptive summarization of the proposed BanglaSER dataset.

Year	2022
Language	Bangla
Dataset type	Acted, Scripted
File Type	Audio only
The file format of audio clips	.WAV
Sampling rate	44.1 kHz
No. of actors	34 (17 males and 17 females)
Age group of actors	19 to 47 years
No. of emotions	5
No. of emotional states	Angry, Happy, Neutral, Sad, Surprise
No. of statements	3
No. of audio clips	1467
Size of the dataset	776.8 MB
Unit level	Sentence
No. of words	11
No. of vowels in the texts (Phonetic)	23
No. of constants in the texts (Phonetic)	24
No. of other phonemes in the texts (Phonetic)	0 Diphthongs and 0 nasalization
The average duration of each clip	3 to 4 s
Utilized Software	Audacity
Duration	1 h 29 min (Approx.)
Human accuracy	80.5% (approx.)

## 2 Experimental Design, Materials and Methods

**Fig. 4:**
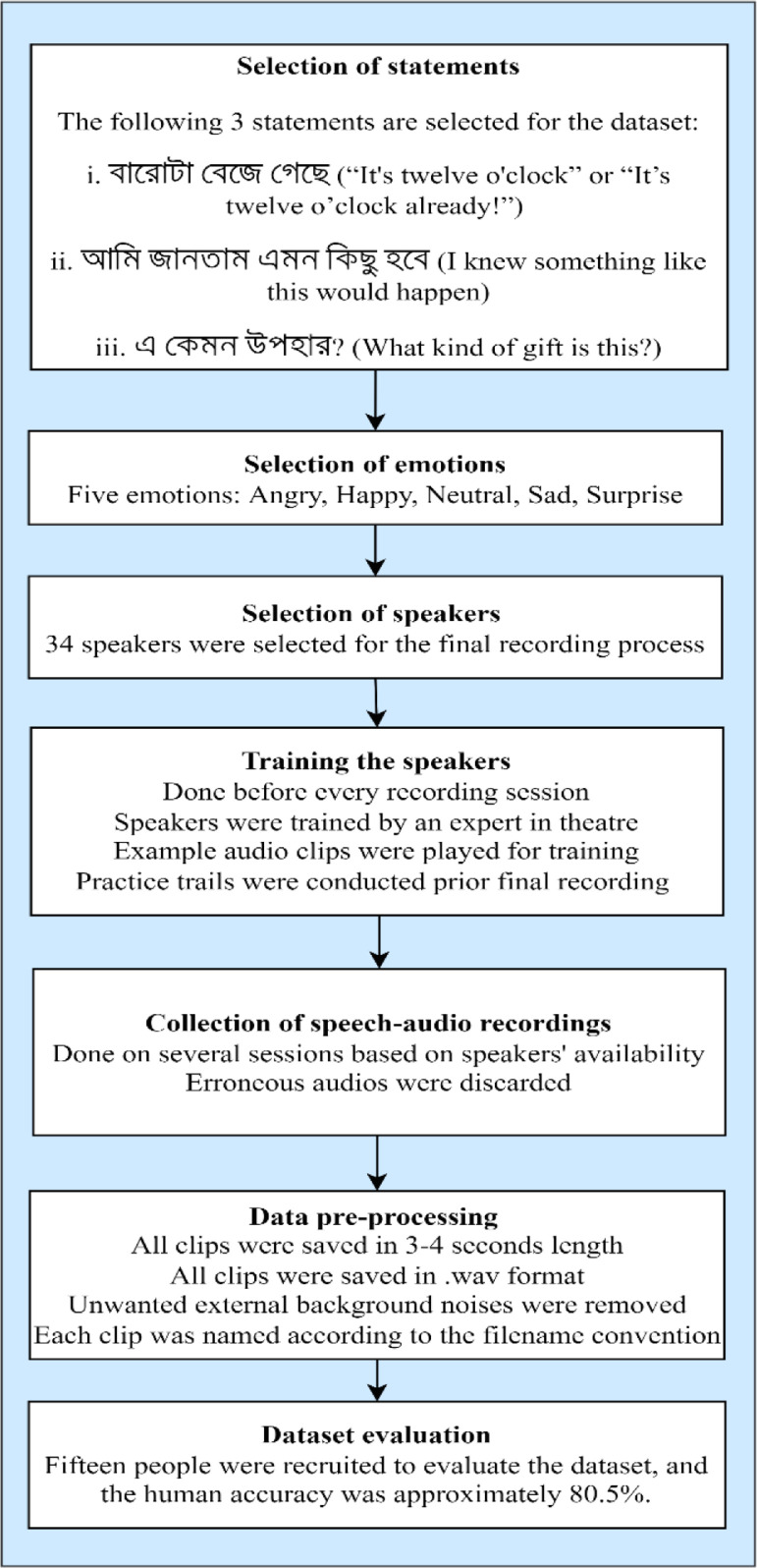
Workflow diagram of the BanglaSER dataset preparation.

The BanglaSER dataset preparation consists of seven steps: statements preparation, emotion selection, speaker selection, training the speakers, voice/audio recordings collection, data pre-processing, and evaluation. This section briefly describes each of these steps to prepare the BanglaSER dataset. The workflow diagram of the BanglaSER preparation is presented in Fig. 4.

### 2.1 Selection of statements

The BanglaSER dataset is created to induce emotional behaviors associated with fixed lexical content yet conveying distinct emotional states, referred to as target statements. We have initially selected ten Bangla statements that will be acted on with five different emotional states. After initial testing of the statements, three statements were then selected out of those ten statements. The final three statements are:

**Table:** 

**Statement 1:** Bangla: English 1: It's twelve o'clock. English 2: It's twelve o'clock already! Phonemic: bAro Ta beje geche. Phonetic: bʌrɔ tʌ bedʒe ɡetʃ^h^e	**Statement 2:** Bangla: English: I knew something like this would happen. Phonemic: Ami jantam emon kichu hobe. Phonetic: ʌmi dʒʌntʌm æmɔn kitʃ^h^ʊ hɒbe.	**Statement 3:** Bangla: ? English: What kind of gift is this? Phonemic: E kemon UpoHar. Phonetic: e kæmɔn ʊpɔhʌr.

### 2.2 Selection of emotions

According to basic emotion theory [14], humans possess a limited set of ``basic'' emotions (e.g., fear, anger, happiness, and sadness). For the BanglaSER dataset, we have selected three of the mentioned rudimentary emotions of the human being, namely anger, happiness, sadness. However, in the existing literature [2,5,6], neutral and surprise emotions are significantly explored too. Initially, we had considered six basic human emotional states: anger, disgust, neutral, happiness, sadness, and surprise. However, due to the quite similar high amplitude and pitch issue of both angry and disgust emotions, emotions of both types quite often overlapped with each other, so we discarded the disgust emotion from this dataset and only kept the angry emotion. As a result, the most clear and obvious emotional states were implicated, resulting in the BanglaSER dataset's five emotions: angry, happy, neutral, sad, and surprise. The dataset maintains a balanced number of recordings in each of these emotions.

### 2.3 Speaker selection

Initially, forty-two randomly selected nonprofessional actors living in Dhaka, Bangladesh, working in various sectors voluntarily participated in the speech-audio recordings. Thirty-four speakers were then selected for the final data collection process aged between 19 and 47 years, with 17 male and 17 female participants equally distributed.

### 2.4 Training the speakers

Speakers were given a detailed briefing about all the statements and emotional states. There are example audio samples for all emotional statements to make them understand. An expert in theatre trained the actors face to face or via video conferencing. During the training phase, actors were informed that they would be allowed as much time as necessary to acquire the proper emotional state and that they would signal their readiness once achieved. After extensive preparation, and when the participants felt prepared, the recordings were collected.

### 2.5 Collection of speech-audio recordings

Each actor provided audio recordings for each of the five emotional states. For three statements, with three repetitions, every actor provided a total of 36 recordings of angry, happy, sad, and surprise emotions. Additionally, out of 36 participating actors, 27 actors provided the neutral emotion recordings for three statements, with three repetitions, resulting in further 27 × 3 × 3 = 243 neutral speech-audio recordings. Data were collected on multiple sessions based on the actor's availability. Due to the COVID-19 restriction issues, data from some actors have been collected through the smartphone's recording application, while the majority of the actors were recorded individually in a quiet, noiseless room, providing a suitable acoustic environment for high-quality speech-audio recordings. The authors discarded erroneous recordings. With the help of an expert in theatre, proper guidelines were given to the participating actors so that accurate emotions can be captured. A condenser microphone (BOYA BY-M1 Omnidirectional lavalier) with an appropriate microphone stand was provided to the speaker for recording. The distance was set at 6 cm between the microphone and actors. We connected the microphone to a USB audio interface. An AKG-K240 studio professional headphone was also connected to the audio interface so that specialists could listen to the audio during recordings. A laptop with an Intel Core i7 processor, Nvidia GTX 1050-Ti graphics card was used with 16 GB RAM as the hardware tools and 64-bit Windows 10 as the operating system. The recordings are processed at a sampling rate of 44.1 kHz. For audio preprocessing, an open-source audio editing software, Audacity (version 2.3.2), was used and any external or unwanted background noises were removed, and given proper filenames to each recording.

### 2.6 Data pre-processing

After collecting all the required audio recordings, each of them was converted to .WAV format. Using the Audacity software, all the recording clips were cut to 3 to 4 s of data. Any external noises were also removed. Each data file was then given a unique filename. Every two-digit numerical identifier defines the level of the factors of a different experiment. The identifiers are ordered: Mode - Statement type – Emotional state - Emotion intensity - Statement number- Repetition – Actor (male-odd, female-even).wav. The filename convention is described in Table 4. For example, the filename “03-01-03-02-02-02-05.wav” refers to: Audio only (03) - Scripted (01) - Angry (03) - Strong intensity (02) – 2nd Statement (02) – 2nd Repetition (02) – 5th Actor, male (05). We have a future plan to increase the dataset by adding the multimodal audio-video mode, additional unscripted/improvised recordings, additional statements, and calm, disgust emotions.

**Table 4:** Description of the filename convention.

Identifier	Meaning
Mode	03 = Audio-only
Statement type	01= Scripted
Emotion	01 = Happy, 02 = Sad, 03 = Angry, 04 = Surprise, 05 = Neutral
Intensity	01 = Normal, 02 = Strong
Statements	01 = English 1: It's twelve o'clock English 2: It's twelve o'clock already! 02 = English: I knew something like this would happen. 03 = ? English: What kind of gift is this?
Repetition	01 = 1st repetition, 02 = 2nd repetition, 03=3rd repetition
Actor	01 = First actor, 02 = Second actor…..., 34 = Thirty-four actor (odd-male, even-female)

### 2.7 Dataset evaluation

The dataset evaluation's primary objective is to determine to what extent an untrained and uninformed listener can properly identify the emotion in recorded audios. A higher emotion recognition rate indicates that the recordings are of higher quality. Recently, researchers have utilized crowdsourcing solutions in order to expand and evaluate the emotional content of SER-related datasets [2,15,16]. The crowdsourcing system facilitates the collection of substantial quantities of annotated data. It has the potential to improve future research outcomes in the areas of customized SER and speaker-agnostic SER. It enables remote involvement and contributions to the dataset's proliferation [16]. However, instead of crowdsourcing, we have chosen 34 participating nonprofessional actors for the data collection process and fifteen human validators, 8 male and 7 female, for a controlled perceptual evaluation over acted read sentences to evaluate the BanglaSER dataset. Each evaluator was either a student or faculty from Stamford University Bangladesh and United International University. A large number of evaluators offer better identification of the perceived emotions. At the time of the evaluation, all individuals were physically and mentally fit and above the age of 19. No evaluator participated in the speech-audio recording sessions. They were all native Bangla speakers who could fluently read, write and interpret the language. The evaluators were not trained on the recordings to eliminate any prejudice in their ability to perceive emotions. We developed a management software for the overall management of the evaluation process of the BanglaSER dataset. Each evaluator evaluated a set of speech-audio recordings in three sessions. Every set consisted of around 98 recordings from all emotional classes. In each session, an evaluator evaluated approximately 33 recordings. We examine the effectiveness of the emotions of the dataset by analyzing the target statements' confusion matrix. Table 5 summarizes the findings, which indicate that in 80.5% of cases, the evaluators correctly perceived (i.e., the emotion selected most often by the evaluators) the target emotional class. The surprise emotional class is the most difficult emotional class to perceive, as it is frequently confused with happy speech. To a degree, the neutral emotional class is mistaken for the sad class. Note that, similar confusion has been seen in prior studies too [2,11]. Recognition rates are highest for angry followed by neutral, happy, sad, and surprise emotions.

**Table 5:** Confusion matrix for the actual versus perceived emotions for target statements during the dataset evaluation process.

		Perceived emotions (80.5%)				
	Category	Angry (%)	Happy (%)	Neutral (%)	Sad (%)	Surprise (%)
**Actual emotions**	Angry	**84.5**	2	0.5	5	8
	Happy	3.5	**80**	3	1.5	12
	Neutral	1	3.5	**81.5**	11.5	2.5
	Sad	2.5	1.5	14	**79.5**	2.5
	Surprise	3	13.5	5	1.5	**77**

## Ethics Statements

Informed consent to release the speech-audio recordings was acquired from each participating actor. Participants were informed that participation was voluntary and that they were free to leave or pause the speech recordings at any time. All data collected from the voluntarily participating actors were anonymized after the full data collection process. It was ensured that any information submitted would be treated confidentially and in an anonymous manner. Each participant has approved the samples post-recording. No ethical approval was required.

## CRediT authorship contribution statement

**Rakesh Kumar Das:** Methodology, Conceptualization, Software, Formal analysis, Data curation. **Nahidul Islam:** Investigation, Conceptualization, Data curation, Software. **Md. Rayhan Ahmed:** Methodology, Validation, Writing – original draft, Supervision, Project administration. **Salekul Islam:** Validation, Writing – review & editing. **Swakkhar Shatabda:** Investigation, Writing – review & editing. **A.K.M. Muzahidul Islam:** Validation, Writing – review & editing.

## Declaration of Competing Interest

The authors declare that they have no known competing financial interests or personal relationships that could have appeared to influence the work reported in this paper.

## Data Availability

BanglaSER: A Bangla speech emotion recognition dataset (Original data) (Mendeley Data).
